# Combined metabolic stress and nitric oxide synthase inhibition induces tissue-specific remodeling across cardiovascular and hematopoietic compartments during obesity

**DOI:** 10.3389/fcvm.2026.1934423

**Published:** 2026-08-31

**Authors:** Julia Drolet, Sergio D. Gomez Navarro, Ian M. Davis, Madison Babcock, Aric Lechner, Norifumi Urao

**Affiliations:** Department of Pharmacology, SUNY Upstate Medical University, Syracuse, NY, United States

**Keywords:** bone marrow remodeling, cardiometabolic disease, heart failure with preserved ejection fraction, hematopoietic niche, obesity

## Abstract

Obesity-associated cardiometabolic disease is characterized by tissue-specific remodeling across cardiovascular, immune, and skeletal tissues. However, the integrated effects of combined metabolic stress and nitric oxide synthase (NOS) inhibition on these organ systems remain incompletely understood. We investigated whether long-term high-fat diet (HFD) feeding combined with NOS inhibition by L-NAME differentially remodels multiple organs during obesity progression. Male C57BL/6J mice were fed chow, HFD (60% kcal from fat), or HFD supplemented with constitutive NOS inhibition by L-NAME (0.5 g/L drinking water) for 15 weeks. HFD increased body weight, cardiac hypertrophy, myocardial fibrosis, and circulating myeloid-cell expansion compared with chow-fed controls. In contrast, L-NAME supplementation did not further increase body weight, cardiac hypertrophy, myocardial fibrosis, or collagen type I/type III ratio. Obesity-associated expansion of circulating neutrophils and inflammatory Ly6C^hi^ monocytes was not observed in HFD + L-NAME mice, and instead, HFD + L-NAME selectively enhanced bone marrow remodeling, characterized by increased marrow adiposity and reduced trabecular bone volume. These findings demonstrate that combined HFD and NOS inhibition does not uniformly exacerbate obesity-induced pathology but instead elicits tissue-specific remodeling across organ systems. Under these experimental conditions, we highlight the bone marrow as a tissue prominently remodeled during combined metabolic stress and NOS inhibition, identifying the hematopoietic niche as a previously underappreciated target of cardiometabolic stress.

## 1 Introduction

Obesity-associated cardiometabolic disease is characterized by heterogenous remodeling across cardiovascular, immune, metabolic, and skeletal systems. Although many obese individuals initially exhibit excess adiposity with preserved organ function (“preclinical” obesity), others progress to “clinical” obesity, characterized by overt complications affecting multiple organ systems. Recent clinical frameworks emphasize that this transition is driven not simply by increasing adiposity but by complex interactions among adipose tissue, the cardiovascular system, immune cells, and stromal niches (1).

Clinical obesity commonly manifests as a multi-organ syndrome involving heart failure with preserved ejection fraction (HFpEF) (2, 3), type 2 diabetes, metabolic dysfunction-associated steatotic liver disease (MASLD) (4), bone fragility (5), and impaired wound healing (6). These complications are linked by chronic low-grade inflammation, metabolic dysregulation, and endothelial and microvascular dysfunction. Obesity-associated HFpEF has emerged as a systemic cardiometabolic syndrome in which metabolic stress, hypertension, microvascular dysfunction, and immune activation impair diastolic reserve and exercise tolerance despite preserved ejection fraction (3, 7, 8). In parallel, clinical studies have identified altered circulating CD34⁺ hematopoietic/progenitor-cell profiles in obesity and metabolic syndrome (9, 10), impaired progenitor-cell mobilization in diabetes (11), chronic perturbation of circulating monocytes and neutrophils in obesity and diabetes (12), and changes in bone marrow adiposity in individuals with obesity and diabetes (13, 14), raising the possibility that obesity progression is accompanied by coordinated remodeling across cardiovascular, immune, metabolic and skeletal compartments.

Rodent models have been indispensable for dissecting the mechanisms linking obesity to organ dysfunction. Conventional diet-induced obesity (DIO) models using high-fat or Western diets in C57BL/6 mice recapitulate many metabolic features of human obesity, including weight gain, insulin resistance, hepatic steatosis (15), mild wound healing delay (16), dysregulated hematopoiesis (17–19), and modest cardiac hypertrophy with preserved ejection fraction and mild diastolic impairment (20). However, these models typically emphasize individual organ systems and do not consistently reproduce the spectrum of multi-organ abnormalities observed in clinical obesity, including the HFpEF phenotype (21–23). To better model obesity-associated HFpEF, several “multi-hit” approaches have been developed. Among them, the high-fat diet plus L-NAME (HFD + L-NAME) model combines metabolic overload with nitric-oxide synthase (NOS) inhibition has been reported to produce a HFpEF-like phenotype with preserved ejection fraction, diastolic dysfunction, pulmonary congestion, cardiac hypertrophy and fibrosis, and reduced capillary density (24). More recently, Filipp et al. demonstrated that this model also remodels the splenic hematopoietic niche through fatty acid-driven macrophage activation, promoting extramedullary myelopoiesis (25). Together, these findings suggest that HFD + L-NAME induces coordinated remodeling beyond the heart. Nevertheless, the integrated effects of combined metabolic stress and NOS inhibition across cardiovascular, hematopoietic, skeletal, and immune compartments remain poorly defined.

In the present study, we used male C57BL/6J mice exposed for 15 weeks to chow, HFD, or HFD + L-NAME to address this gap. We test the hypothesis that combined metabolic stress and NOS inhibition would differentially remodel cardiovascular, immune, and skeletal compartments compared with HFD alone, by simultaneously quantifying body and organ weights (heart, liver, spleen), peripheral myeloid-cell populations, bone-marrow histomorphometry (trabecular bone volume and marrow adipocytes) and cardiac fibrosis. By situating our findings within the context of existing HFD, genetic, and HFpEF models, we sought to determine how combined metabolic stress and NOS inhibition differentially remodels multiple organs during obesity progression in animal models.

## 2 Methods

### 2.1 Animals

Protocols for animal experiments were conducted with approval from the Institutional Animal Care and Use Committees at Upstate Medical University and in accordance with National Institutes of Health guidelines for the use of laboratory animals. Animals were housed in a 12hr light/dark cycle and temperature-controlled facility. 6-8week old C57Bl6/J (Jackson Laboratories) were fed a high-fat diet (HFD) (60% kcal fat; Research Diets; D12492) with N^[w]^-nitro-l-arginine methyl ester (L-NAME) (0.5 g/L, Sigma Aldrich) supplemented drinking water ad libitum for 15 weeks (24). Control mice were fed normal chow diet (Lab Diet; 5008). Food and water intake were not measured. No mice were excluded from analysis unless noted.

### 2.2 Quantification of fibrotic area in heart tissue

Deparaffinized transverse heart sections (8 µm thickness) were stained with picrosirius red (ab150681, abcam) for 60 minutes followed by rinsing in acetic acid. Images of picrosirius red stained heart sections acquired in an ImageXpress Pico microscope (Molecular Devices) were processed in Fiji to determine the percent of fibrotic tissue area.

To determine the ratio of collagen type I to type III in picrosirius red stained heart sections, polarized light images were acquired with a THUNDER DM6 B upright light microscope (Leica Microsystems). In Fiji, individual color channels were split, and the area of collagen type I (red channel) and type III (green channel) were quantified in the left ventricular area and in vascular regions after the exclusion of background signal by the Renyi Entropy threshold. Image acquisition and analysis were performed blinded.

### 2.3 Flow cytometry

Peripheral blood was collected retro-orbitally into an EDTA coated tube. Following red blood cell lysis and pre-incubation with FC block, blood cells were stained with anti- CD45 PerCP (30-F11, 103129, BioLegend), CD11b BV605 (M1/70, 101257, BioLegend), Ly6G BV711 (1A8, 127643, BioLegend), CD115 PE-Dazzle594 (AFS98, 135527, BioLegend), and Ly6C BV785 (HK1.4, 128041) antibodies at 1:200 concentration. Live cells were identified with Live/Dead Fixable Blue Dead Cell Stain (L23105, ThermoFisher). Flow cytometry was performed on Cytek Aurora and analyzed with FlowJo software v.10.10.0 (BD Biosciences). Fluorescence compensation was performed using antibody capture compensation beads stained individually with each fluorochrome-conjugated antibody. Unstained cells were used to establish background fluorescence, and dead cells were excluded using Live/Dead Fixable Blue viability dye. Gating was performed using sequential exclusion of debris, doublets, and dead cells, followed by identification of leukocyte populations as shown in Supplementary Figure S1.

### 2.4 Bone histological analysis

Formalin fixed femurs were decalcified in EDTA/Sucrose Decalcifying Solution (1048B, Newcomer Supply) and embedded in paraffin for coronal sectioning at a thickness of 8 µm. Deparaffinized sections were stained with hematoxylin and eosin (H&E) (H-3502, Vector Laboratories). Bone marrow adipocyte number was quantified within a 1.5mm × 1.5 mm region including bone marrow tissue extending from the distal growth to 1600 µm above the growth plate. Trabecular bone area was quantified in a 2.25 mm^2^ region located 0.5 mm above the distal growth plate in the most medial sections of each mouse femur. Only slides that displayed continuation from the metaphysis to the diaphysis were included in analysis resulting in exclusion of 5 Control, 4 HFD, and 4 HFD + L-NAME sections. Images of H&E-stained bones were acquired in an ImageXpress Pico microscope (Molecular Devices) and blinded quantification was performed in Fiji.

## 3 Results

### 3.1 Combined metabolic stress and NOS inhibition differentially affects organ remodeling

**Figure 1:**
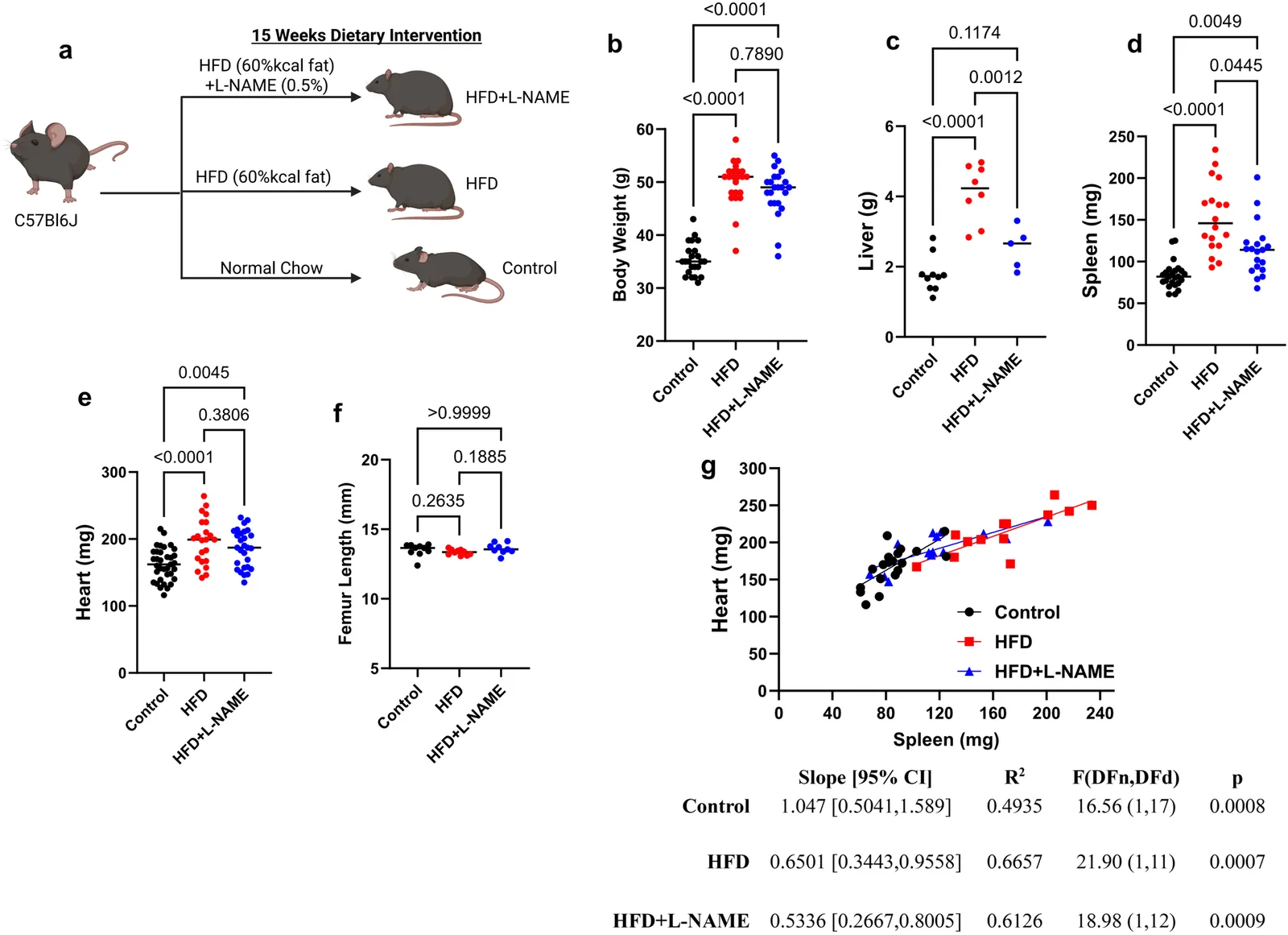
Combined metabolic stress and NOS inhibition differentially affects organ remodeling. **(a)** Experimental design. C57BL/6J mice were fed chow, HFD, or HFD plus L–NAME for 15 weeks. Created with BioRender.com. **(b)** Body weight measured after 15 weeks of diet treatment in control, HFD, and HFD + L-NAME groups. (total *N* = 23 from 4 cohorts of 4–5 animals per group) **(c)** Liver weights (total *N* = 5–10 animals from 2 cohorts of 2–5 animals per group), **(d)** spleen weights (*N* = 18–23 animals from 4 cohorts), **(e)** total heart weights (total *N* = 22−35 animals from 6 cohorts of 3–6 animals per group), **(f)** and femur length (total *N* = 9–10 animals from 2 cohorts of 4–5 animals per group) **(g)** Correlation between spleen and heart weight (total *N* = 13–19 from 4 cohorts of 3–5 animals per group). Each point represents one biological replicate. Statistical significance was determined by Kruskal-Wallis with Dunn's multiple comparisons test for **(b,d,e,f)**. For data passing Shapiro-Wilk test for normality, One-Way ANOVA with Tukey's multiple comparisons test was used.

To determine how metabolic stress and NOS inhibition influences organ remodeling during obesity progression, mice were fed a HFD (60% kcal from fat) supplemented with L-NAME in the drinking water (HFD + L-NAME mice) for 15 weeks (Figure 1a). After 15 weeks, HFD significantly increased body weight relative to chow-fed controls, and L-NAME did not further affect weight gain (Figure 1b). Consistent with prior studies demonstrating that generalized body-mass gain by chronic HFD exposure is accompanied by the enlargement of metabolic organs (26, 27), HFD also increased liver, spleen, and heart weight, consistent with obesity-associated organ enlargement (Figures 1c–e). Intriguingly, L-NAME supplementation selectively reduced liver and spleen weight while having little effect on cardiac hypertrophy, indicating that metabolic stress and NOS inhibition differentially influences organ remodeling rather than uniformly exacerbating obesity-induced changes. Notably, there were no differences in femur length across any group (Figure 1f), indicating that organ remodeling was not influenced by skeletal growth. Across all animals, spleen weight positively correlated with heart weight (Figure 1g), suggesting that splenic enlargement accompanies cardiac remodeling during obesity progression. Together, these findings demonstrate that L-NAME selectively modifies obesity-induced organ remodeling, reducing liver and spleen enlargement without further affecting body weight or cardiac hypertrophy.

### 3.2 Cardiac fibrosis and collagen composition are comparable between HFD and HFD+L-NAME mice

**Figure 2:**
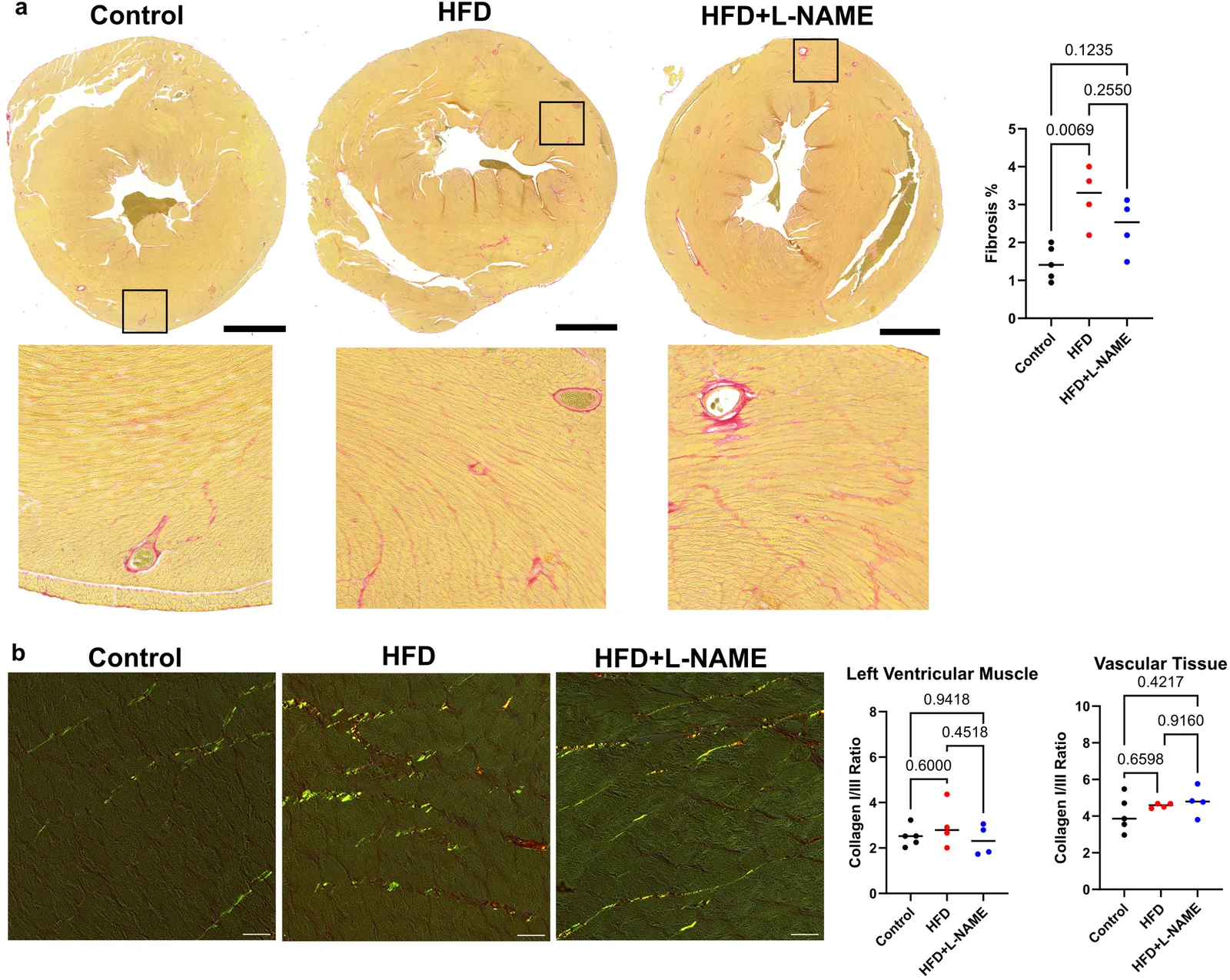
Cardiac fibrosis and collagen composition are comparable between HFD and HFD + L-NAME mice. **(a)** Representative picrosirius red-stained transverse heart sections and quantification of fibrotic area. Scale bar = 200 µm. **(b)** Polarized light images of picrosirius red stained heart sections were quantified for the ratio of collagen type I (red) to collagen type III (green). Scale bar = 25μm. Data was quantified from *N* = 4–5 sections per group obtained from individual mice. Assumption of normality was tested by Shapiro–Wilk test and statistical significance was determined by One-Way ANOVA with Tukey's multiple comparisons test.

Because heart weight was similarly increased in HFD and HFD + L-NAME mice (Figure 1e), we next determined whether combined HFD and NOS inhibition further altered myocardial remodeling by quantifying cardiac fibrosis and collagen composition. Picrosirius red staining demonstrated that HFD increased myocardial fibrosis relative to chow-fed controls; however, fibrosis was not further increased by L-NAME supplementation (Figure 2a). We next assessed the ratio of collagen type I to type III, a histological feature associated with increased myocardial stiffness in HFpEF (28, 29). Neither HFD alone nor HFD + L-NAME altered the collagen type I/type III ratio compared with controls, and no difference was detected between the two HFD groups (Figure 2b). Together, these findings suggest that the cardiac response to combined metabolic stress and NOS inhibition may plateau under our experimental conditions, prompting us to examine whether immune and hematopoietic compartments exhibit similar or divergent responses.

### 3.3 Combined metabolic stress and NOS inhibition alters obesity-induced circulating myeloid-cell expansion

**Figure 3:**
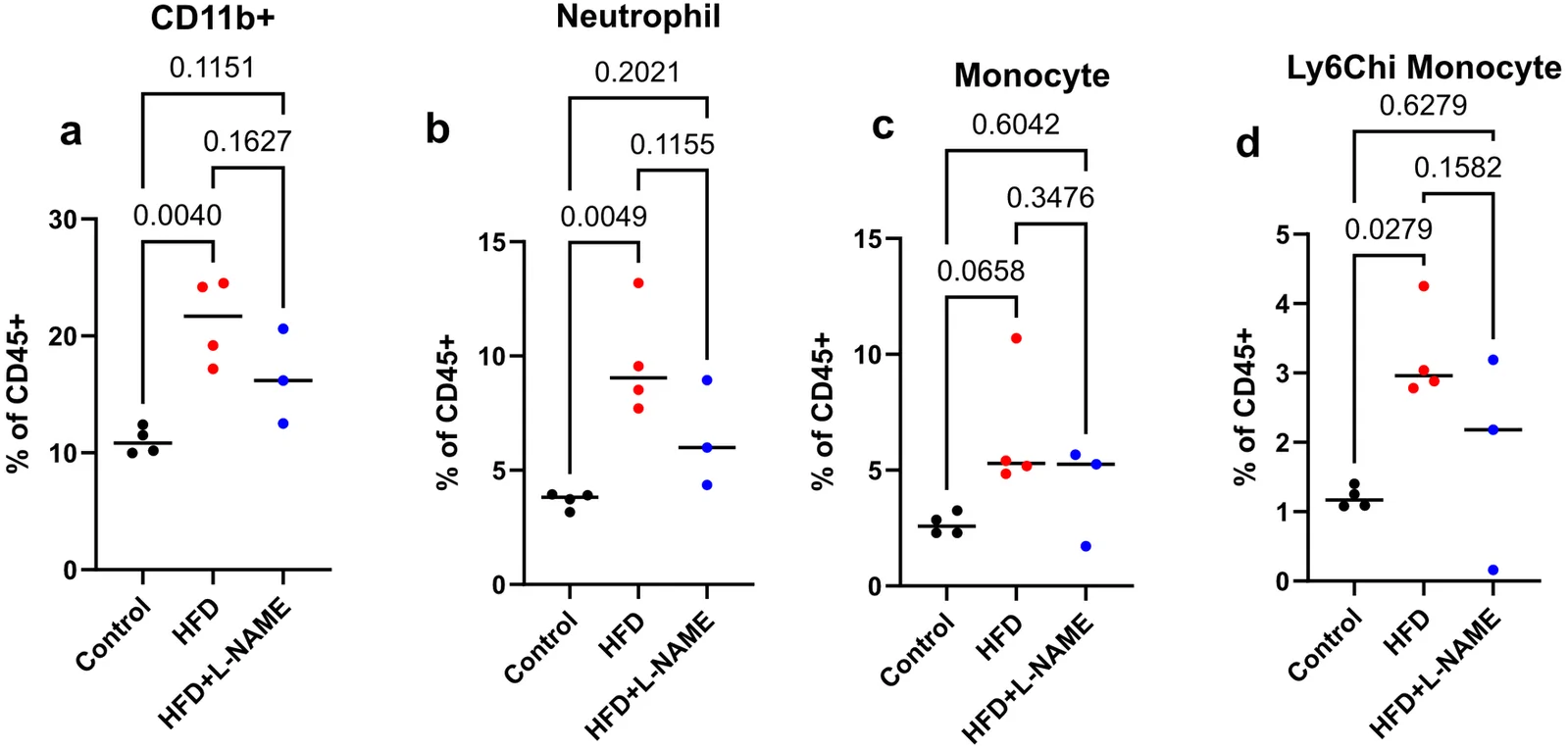
Combined metabolic stress and NOS inhibition alters obesity-induced circulating myeloid-cell expansion. Frequencies of **(a)** CD11b^+^ myeloid cells, **(b)** Ly6G^+^ neutrophils, **(c)** monocytes, and **(d)** Ly6C^hi^ inflammatory monocytes in peripheral blood collected from mice after 15 weeks of dietary intervention. *N* = 3–4 individual mice per group with each point representing one individual biological replicate. Normality was determined by Shapiro–Wilk test and statistical significance was determined by One-Way ANOVA with Tukey's multiple comparisons test.

Because HFD + L-NAME mice exhibited reduced spleen weight relative to HFD mice, we next determined whether this was accompanied by changes in circulating myeloid-cell populations. Consistent with previous reports demonstrating that HFD promotes inflammatory myelopoiesis (17, 30), HFD increased the proportion of circulating CD11b + myeloid cells (Figure 3a). In contrast, HFD + L-NAME mice exhibited levels comparable to chow-fed controls. Similarly, circulating Ly6G⁺ neutrophils were increased in HFD mice but not in HFD + L-NAME mice (Figure 3b). Although total circulating monocyte frequencies were not significantly altered among groups (Figure 3c), HFD selectively increased the proportion of inflammatory Ly6C^hi^ monocytes compared to control, an effect that was not observed following L-NAME supplementation (Figure 3d). These findings highlight that, combined HFD and NOS inhibition did not produce significant expansion of circulating myeloid-cell populations relative to chow-fed controls in contrast to HFD alone, where the expansion of circulating myeloid-cell populations, driven by an increase in neutrophils and inflammatory monocytes, was observed. The discordance between circulating myeloid-cell expansion and organ remodeling prompted us to examine whether the bone marrow itself exhibited evidence of structural remodeling in response to combined metabolic stress and NOS inhibition.

### 3.4 Bone marrow remodeling is selectively enhanced by combined metabolic stress and NOS inhibition

**Figure 4:**
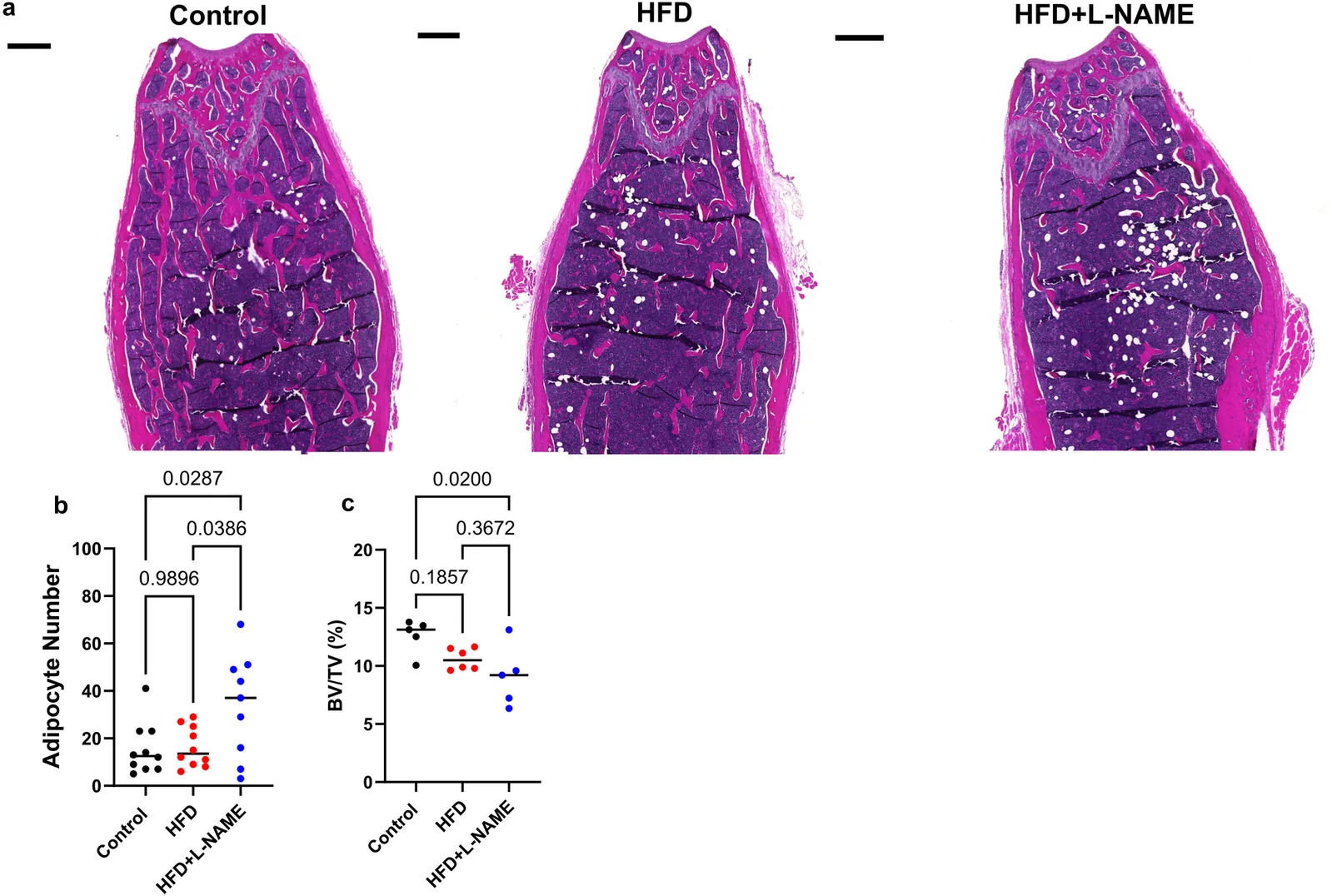
Bone marrow remodeling is selectively enhanced by combined metabolic stress and NOS inhibition. **(a)** Distal femur sections stained with hematoxylin and eosin (H&E); scale bar=500um. **(b)** Adipocyte numbers were quantified in the bone marrow across the region extending from the growth plate to 1600 μm. Data was quantified from *N* = 9–10 sections per group obtained from two cohorts of 4–5 individual mice per group. **(c)** Quantification of trabecular bone volume (BV) expressed as a percentage of the total volume (TV) measured within a 1.5 × 1.5 mm region above the growth plate. Normality was determined by Shapiro–Wilk test and statistical significance was determined by One-Way ANOVA with Tukey's multiple comparisons test.

Because HFD and NOS inhibition did not change circulating myeloid-cell expansion despite reduced spleen size, we next examined whether structural remodeling occurred within the bone marrow microenvironment. Bone marrow adipocytes and trabecular bone are integral components of the hematopoietic niche and undergo remodeling during obesity. During weight gain, systemic adipogenesis is accompanied by a parallel expansion of bone marrow adipocytes which are located proximal to hematopoietic tissues (31) and secrete factors such as adiponectin (32) and stem cell factor (31) which regulate hematopoietic stem cell (HSC) survival and self-renewal capacity respectively. In HFD mice, enhanced adipocyte expansion at the expense of osteoblast differentiation leads to a reduction in trabecular bone volume (26). Because obesity alters the bone marrow microenvironment, we next quantified trabecular bone volume and marrow adiposity in distal femurs. HFD alone produced only modest changes in bone marrow architecture, whereas HFD + L-NAME markedly expanded the number of adipocytes in the bone marrow compartment (Figures 4a, b). The expansion of bone marrow adipocytes in HFD + L-NAME mice was accompanied by a loss in trabecular bone volume compared to control mice (Figure 4c), consistent with enhanced remodeling of the hematopoietic niche. Intriguingly, HFD + L-NAME mice exhibited an increase in marrow adiposity compared to HFD feeding alone, whereas trabecular bone volume loss was not worsened by L-NAME addition, indicating that NOS inhibition strikingly affects marrow adiposity. Together, these data demonstrate that combined HFD and NOS inhibition selectively amplifies bone marrow remodeling despite relatively modest additional effects on cardiac remodeling or circulating myeloid-cell expansion. Collectively, these findings identify the bone marrow as a particularly responsive target of combined metabolic stress and NOS inhibition, supporting the concept that cardiometabolic remodeling is accompanied by tissue-specific remodeling rather than uniform exacerbation of pathology.

## 4 Discussion

In this study, we investigated how combined metabolic stress and NOS inhibition influences multi-organ remodeling during obesity progression. As expected, HFD feeding increased body weight, organ weight, circulating myeloid cells, and myocardial fibrosis. However, the addition of L-NAME did not uniformly amplify these obesity-associated changes. Although cardiac hypertrophy and fibrosis were not further exacerbated and circulating myeloid-cell expansion was not observed, HFD + L-NAME markedly enhanced bone marrow remodeling, characterized by increased marrow adiposity and reduced trabecular bone volume compared to chow-fed controls. These findings demonstrate that combined metabolic stress and NOS inhibition elicits tissue-specific responses rather than uniformly worsening obesity-induced pathology, identifying the bone marrow as a particularly responsive target of impaired NOS inhibition under our experimental conditions.

Our results align partially with the original description of the HFD + L-NAME “two-hit” HFpEF model by Schiattarella et al (24). In their study, the combination of HFD and NOS inhibition elicited hallmark HfpEF features—including diastolic dysfunction, pulmonary congestion, reduced exercise tolerance, and myocardial capillary rarefaction in C57BL/6N strain. Similar cardiac phenotypes have subsequently been reported in other HFD + L-NAME studies in C57BL/6J mice (25, 33), whereas conventional HFD alone induced diastolic dysfunction and cardiac hypertrophy in C57BL/6J mice as early as 1 month (20). In direct comparison, both similar and less pronounced cardiac phenotype in C57BL/6J compared to C57BL/6N after HFD-L-NAME have been reported (34, 35). Although we observed increased cardiac hypertrophy and fibrosis in both HFD groups, L-NAME did not further augment these changes under our experimental conditions. Although NOS inhibition did not further exacerbate myocardial hypertrophy or fibrosis in our model, cardiac remodeling is regulated by multiple conserved intracellular signaling pathways that integrate hypertrophic and profibrotic stimuli. Among these, the PTEN/AKT/Mtor pathway has been shown to play an important role in coordinating pathological cardiac hypertrophy and fibrosis in response to cardiovascular stress (36), underscoring the complexity of signaling networks governing myocardial adaptation. In addition, our small male C57BL/6N cohort did not show a clear increase in heart weight (Supplementary Figure S2). Therefore, these differences and inconsistencies may reflect variable degrees of NOS inhibition induced by L-NAME, or housing environment factors influencing microbiome or stress levels. Notably, most previous studies evaluated cardiac function after approximately five weeks of HFD + L-NAME exposure (24, 25, 33), whereas our analyses were performed after 15 weeks. It is possible that prolonged metabolic stress alters the trajectory of tissue remodeling, such that early inflammatory and cardiovascular responses become less distinguishable while tissue-specific structural remodeling emerges as a more prominent feature. Under this framework, the differences between our findings and earlier studies may reflect temporal evolution of the HFD + L-NAME phenotype rather than conflicting biological mechanisms. Longitudinal studies incorporating both early and late time points will be needed to define the sequence of cardiovascular, immune, and hematopoietic remodeling during disease progression.

Recent mechanistic findings from Filipp et al. (25) provide important context for our observations by demonstrating that HFD + L-NAME remodels extracardiac immune–hematopoietic networks, promoting splenic hematopoiesis and systemic inflammation through metabolically activated red pulp macrophages. Although our study did not evaluate splenic hematopoiesis directly, our finding that the HFD induced expansion of circulating neutrophils and inflammatory Ly6C^hi^ inflammatory monocytes was not observed in HFD + L-NAME mice highlights the complexity of immune regulation in this model. Importantly, our measurements were performed after 15 weeks of dietary intervention, whereas Filipp et al. examined mice after five weeks, raising the possibility that immune remodeling evolves dynamically over time in response to chronic metabolic stress and NOS inhibition. Because the differences between HFD and HFD + L-NAME mice did not reach statistical significance, the mechanisms underlying the altered pattern of circulating myeloid-cell populations remain unclear. Future studies should determine whether nitric oxide signaling influences myeloid-cell dynamics through regulation of bone marrow egress, peripheral clearance, or tissue recruitment during chronic metabolic stress. These observations prompted us to consider whether structural remodeling within the bone marrow might be more responsive to prolonged metabolic stress and NOS inhibition than changes in circulating immune cells.

In contrast to the relatively modest additional changes observed in the heart and circulating myeloid compartment, the bone marrow exhibited striking structural remodeling following HFD + L-NAME treatment. Among the organs examined, the bone marrow exhibited striking sensitivity to combined metabolic stress and NOS inhibition, as evidenced by marked expansion of marrow adipocytes together with reduced trabecular bone volume. Because the bone marrow functions as both a skeletal and hematopoietic organ, these structural changes may have important implications for hematopoietic niche function in obesity. Previous studies have demonstrated that impaired nitric oxide signaling disrupts osteoblast maturation and promotes marrow adiposity, supporting a role for endothelial NOS in maintaining bone homeostasis (37). Our findings extend these observations by suggesting that NOS inhibition preferentially remodels the bone marrow microenvironment during chronic metabolic stress. Although we did not directly assess hematopoietic stem-cell function or niche composition, these structural changes are consistent with the possibility that alterations in the marrow microenvironment contribute to the heterogeneous immune responses observed during obesity progression. Although we did not directly assess cardiac function or hemodynamics, the bone marrow remodeling observed in HFD + L-NAME mice may have functional implications beyond skeletal tissues. Bone marrow adipocytes and trabecular bone are integral components of the hematopoietic stem cell niche, and alterations in their balance can influence hematopoietic homeostasis and systemic inflammatory responses. Consistent with previous studies linking obesity-associated hematopoietic remodeling to chronic inflammation, our findings suggest that remodeling of the bone marrow niche may indirectly contribute to cardiovascular disease progression. These findings suggest that structural remodeling of the bone marrow may represent a sensitive indicator of chronic metabolic and vascular stress under certain experimental conditions.

The tissue-specific responses observed across the heart, peripheral blood, and bone marrow highlight an important feature of cardiometabolic remodeling. Individual organs appear to exhibit distinct thresholds of susceptibility that likely reflect differences in local vascular, stromal, and immune microenvironments. These differences likely reflect intrinsic variation in cellular metabolism and local microenvironment. In the heart, chronic lipid overload drives metabolic remodeling through dynamic interactions between mitochondrial function and fatty acid metabolism (38), whereas the bone marrow niche is additionally shaped by marrow adipocytes, stromal cells, and hematopoietic cells. Such metabolic specialization may contribute to the differential tissue responses observed in our study. This concept parallels the clinical heterogeneity of obesity, in which patients with similar degrees of adiposity frequently develop markedly different cardiovascular, skeletal, or immunometabolic complications. We have previously shown that wound closure is impaired in both HFD alone and HFD + L-NAME mouse models compared to normal/control chow diets, along with increased myelopoiesis, myeloid bias, macrophage dysfunctions, and associated epigenetic and metabolic alterations (16, 30, 39). Together with the present findings, these observations support the concept that cardiometabolic remodeling during obesity progression involves coordinated but tissue-specific remodeling across multiple organ systems rather than parallel deterioration of all tissues.

Several limitations should be considered when interpreting these findings. First, cardiac function and blood pressure were not directly assessed, preventing definitive evaluation of HFpEF severity in our cohort. Second, immune phenotyping was limited to circulating leukocyte populations, and we did not directly examine bone marrow hematopoietic stem and progenitor cells or splenic hematopoiesis. Third, our analyses were performed at a single late time point following 15 weeks of dietary intervention, precluding assessment of the temporal sequence of cardiac, immune, and bone marrow remodeling. Next, our study did not include an L-NAME chow fed group, limiting the interpretation of the independent effect of L-NAME versus the effect of the interaction of combined HFD feeding with L-NAME. Finally, only young adult male mice were studied, and additional investigations should determine whether similar tissue-specific responses occur in females, aged animals, and other models of metabolic disease (16, 30, 39, 40). Because HFD substantially alters body weight, normalization of organ weights to body mass should be interpreted with caution in obesity studies (Supplementary Figure S3) (41), as increases in adiposity may obscure organ-specific remodeling. We therefore presented absolute organ weights and evaluated femur length as an indicator of skeletal growth. Longitudinal studies integrating functional cardiovascular measurements with detailed characterization of hematopoietic niches will help define how metabolic stress and NOS inhibition drive the progression of obesity-associated cardiometabolic disease. More broadly, our findings support the concept that obesity induces organ-specific remodeling rather than uniform pathological progression. As metabolic therapies, including GLP-1 receptor agonists, continue to demonstrate cardiovascular benefit in both clinical and human genetic studies (42), a better understanding of tissue-specific remodeling may facilitate the development of organ-targeted therapeutic strategies for obesity-associated cardiometabolic disease.

## Data Availability

The original contributions presented in the study are included in the article/Supplementary Material, further inquiries can be directed to the corresponding author.
